# Comparison of the Antioxidant Potency of Four Triterpenes of *Centella asiatica* against Oxidative Stress

**DOI:** 10.3390/antiox13040483

**Published:** 2024-04-18

**Authors:** Jinyeong Lim, Hana Lee, Seonghwa Hong, Junsoo Lee, Younghwa Kim

**Affiliations:** 1 Department of Food Science and Biotechnology, Chungbuk National University, Cheongju 28644, Republic of Korea; jinyglim@gmail.com (J.L.); dlgksk0514@naver.com (H.L.); tjd3465@naver.com (S.H.); 2 Department of Food Science and Biotechnology, Kyungsung University, Busan 48434, Republic of Korea

**Keywords:** *Centella asiatica*, triterpenes, antioxidant activity, oxidative stress

## Abstract

We comparatively evaluated the antioxidant properties of key triterpenes from *Centella asiatica*, including asiatic acid (AA), asiaticoside, madecassic acid, and madecassoside, in several cell types, including skin fibroblasts, macrophages, hepatocytes, and endothelial cells, under conditions promoting oxidative stress. AA conferred the highest viability on Hs68 cells exposed to ultraviolet B (UVB) irradiation. Triterpene pretreatment attenuated the UVB-induced generation of reactive oxygen species (ROS) and malondialdehyde (MDA), as well as the UVB-induced depletion of glutathione (GSH) in skin fibroblasts. AA most potently inhibited UVB-induced MMP generation, resulting in increased intracellular collagen levels. Pretreatment with triterpenes, particularly AA, significantly improved cell viability and attenuated TBHP-induced levels of ROS, alanine aminotransferase, and aspartate aminotransferase in HepG2 cells. Triterpenes attenuated ROS levels and reduced MDA and GSH expression in EA.hy926 cells. In RAW264.7 macrophages, production of nitric oxide, tumor necrosis factor-α, and interleukin-6 (indicators of LPS-induced oxidative damage) was significantly reduced by treatment with any of the triterpenes. Statistical analyses of triterpene biological activities using principal component analysis and hierarchical clustering revealed that AA exerted the greatest overall influence and showed remarkable activity in Hs68 and HepG2 cells.

## 1. Introduction

For centuries, *Centella asiatica* L., commonly referred to as Pegaga or Gotu kola, has served as a medicinal herb in China, India, and various other regions of Asia [1]. Moreover, it is commonly used as an herbal medicine but can be eaten fresh in salads, cooked as a vegetable, or blended into drinks [2]. Numerous studies have reported that the main active components of *C. asiatica* are pentacyclic triterpenes. *C. asiatica* extract contains four major triterpenoids: asiatic acid (AA), madecassic acid (MA), asiaticoside (AD), and madecassoside (MD) [3]. Additionally, it contains high levels of total phenolic and flavonoid compounds, including quercetin, kaempferol, catechin, rutin, apigenin, and naringin [4]. The triterpene compounds present in *C. asiatica* mainly consist of glycosides (such as AD and MD), commonly known as saponins, and their aglycone counterparts (such as AA and MA), referred to as sapogenins. AA and MA are biologically active compounds. AD and MD may thus exert their biological activities primarily through conversion into aglycones [5]. These sugars are cleaved in the intestine by enzymes, thereby enabling the absorption of the aglycones. In most studies of the composition of triterpenes in *C. asiatica* leaves, glycoside content has been significantly higher than aglycone content [6]. Sun et al. [3] reported that AA confers potential therapeutic benefits in addressing a wide range of conditions, including cognitive impairment, Alzheimer’s disease, obesity, transverse aortic constriction, atherosclerosis, liver fibrosis, hepatocellular carcinoma, and lung cancer. AD has demonstrated positive therapeutic effects in conditions including Alzheimer’s disease, skin wounds, inflammation, and pulmonary hypertension. In the same study, MA exhibited potential therapeutic effects in ischemic retinopathy, and MD showed promise in treating osteoporosis and acne [3].

Oxidative stress is associated with many pathological conditions and diseases [7]. Oxidative stress in the skin plays a major role in aging and can cause cell and tissue damage [8,9]. Inflammation triggers elevated levels of reactive oxygen species (ROS), contributing to the onset of oxidative stress [10]. Several studies have linked ROS to metabolic disorders, including cancer, insulin resistance, diabetes, cardiovascular diseases, and atherosclerosis [11]. Antioxidants remove free radicals from cells, thereby preventing or minimizing oxidative damage [12]. Given the growing demand for antioxidants and their significance in daily life, the quest for potent, safe, natural compounds with antioxidant properties has garnered increasing participation. The pharmacological effects of *C. asiatica* triterpenes on various diseases have been studied, but no comprehensive comparison of the antioxidant activities of its four main components has been performed. In this study, we compared the antioxidant capacities of AA, AD, MA, and MD in skin fibroblasts, macrophages, hepatocytes, and endothelial cells under oxidative stress.

## 2. Materials and Methods

### 2.1. Chemicals

**Figure 1:**
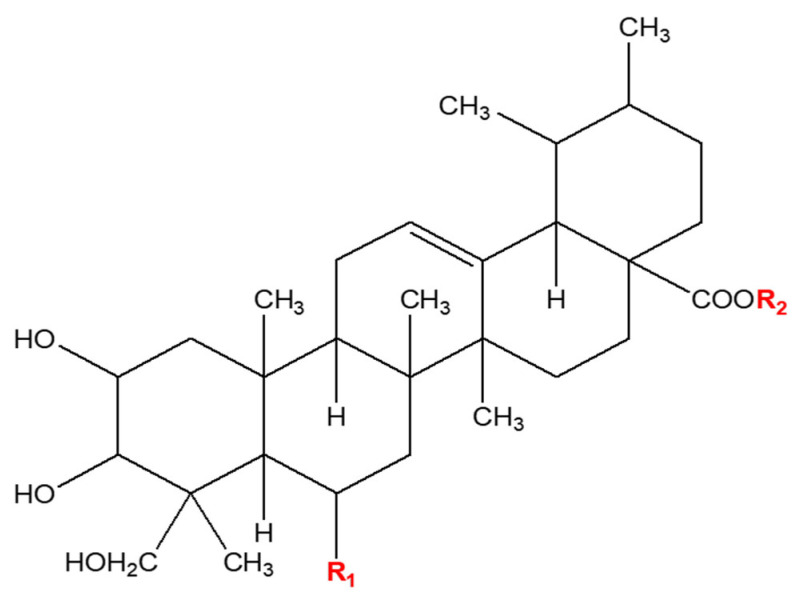
Structures of asiaticoside, madecassoside, asiatic acid, and madecassic acid.

Four triterpenoids (Figure 1), AA, AD, MA, and MD, were obtained from Sigma Chemical Co. (St. Louis, MO, USA). Thiobarbituric acid (TBA), trichloroacetic acid, reduced glutathione (GSH), β- nicotinamide adenine dinucleotide phosphate (NADPH), glutathione reductase (GR), 5-sulfosalicylic acid dihydrate (SSA) ethylene diamine tetra acetic acid (EDTA), 5,5′-dithiobis-2-nitrobenzoic acid (DTNB), Griess reagent, hydrogen peroxide (H_2_O_2_), lipopolysaccharide (LPS), *tert*-butyl hydroperoxide (TBHP), dimethyl sulfoxide (DMSO), 3-(4,5-dimethylthiazol-2-yl)-2,5-diphenyltetrazolium bromide (MTT), and 2′,7′-dichlorofluorescein diacetate (DCFH-DA) were purchased from Sigma Chemical Co. Dulbecco’s modified Eagle’s medium (DMEM), penicillin–streptomycin antibiotic solution, and fetal bovine serum (FBS) were obtained from Gibco BRL (Gaithersburg, MD, USA).

### 2.2. Hs68 Cell Culture

#### 2.2.1. Cell Culture and Cytotoxicity

Hs68 human skin fibroblasts were obtained from the American Type Culture Collection (Manassas, VA, USA). Cells were grown in DMEM containing 10% heat-inactivated FBS, 100 U/mL penicillin, and 100 µg/mL streptomycin, and incubated in a humidified atmosphere containing 5% CO_2_ at 37 °C. Cell viability was determined using a colorimetric MTT assay. Hs68 cells were seeded in 96-well plates at a density of 5.0 × 10^4^ cells/well. After 24 h, cells were pretreated with four triterpenes (10 μM) in a serum-free culture medium for 24 h. Cells were then irradiated with ultraviolet B (UVB) (30 mJ/cm^2^) using a UVB lamp (Sankyo Denki Lamps, GL20SE, Marine, Japan). Radiation intensity was monitored using a UV light meter (LT Lutron, UV-340A, Taipei, Taiwan). After irradiation, cells were treated with compounds in serum-free media for an additional 24 h. Following treatment, MTT solution was added to each well, and cells were incubated at 37 °C for 2 h. After incubation, the culture medium was removed, and DMSO was added to dissolve the formazan. The optical density of each well was measured at 550 nm using a microplate reader (BioTek Instruments, Winooski, VT, USA).

#### 2.2.2. Measurement of Intracellular Reactive Oxygen Species Production

ROS generation was quantified as previously described [13]. Hs68 cells were seeded in black 96-well plates at a density of 5.0 × 10^4^ cells/well. After 24 h, the cells were pretreated with triterpenes (10 μM) in a serum-free culture medium for 24 h. Subsequently, cells were irradiated with UVB (30 mJ/cm^2^) as mentioned above. After irradiation, cells were treated with compounds in serum-free media for an additional 30 min. Cells were then treated with 25 μM DCFH-DA. Fluorescence intensity was assessed using a fluorescence spectrophotometer (Perkin-Elmer, Norwalk, CT, USA) at 10 min intervals over 2 h. Excitation wavelength was set at 485 nm, and emission was detected at 530 nm.

#### 2.2.3. Measurement of Reduced Glutathione and Lipid Peroxidation

Hs68 cells were seeded in a 100 mm dish at a density of 8.0 × 10^4^ cells per well. Cells were then pretreated with compounds (10 μM) and stimulated with UVB radiation, as described above. Cells were harvested and lysed using a Vibra-Cell VCX 750 sonicator (Sonics & Materials Inc., Newtown, CT, USA). Supernatants were immediately used to measure reduced glutathione (GSH) and lipid peroxidation. GSH levels were determined according to the method described by Baker with certain modifications [14]. GSH concentration in cell lysates was determined by referencing a standard curve and is described as nanomoles of GSH per mg of protein.

Lipid peroxidation was determined by levels of malondialdehyde (MDA) measured in the supernatants using a thiobarbituric acid-reactive-substance assay [15]. The absorbance of the supernatant was measured at 535 nm. The results were expressed as nmol of MDA/mg of protein using a molar extinction coefficient of 1.56 × 10^5^ M/cm.

#### 2.2.4. Measurement of Matrix Metalloproteinase-1 and Matrix Metalloproteinase-3

The concentrations of matrix metalloproteinase (MMP)-1 and MMP-3 in culture supernatants were determined using human MMP-1 and MMP-3 ELISA kits (Merck & Co. Inc., Whitehouse Station, NJ, USA) according to the manufacturer’s protocol.

#### 2.2.5. Measurement of Collagen Contents

Hs68 cell collagen content was assessed using a Sircol soluble collagen assay kit (Bioclolor, Belfast, UK). Absorbance was measured at 555 nm using a microplate reader (BioTek Instruments).

### 2.3. HepG2 Cell Culture

#### 2.3.1. Cell Culture and Cytotoxicity

HepG2 cells were seeded in 96-well plates at a density of 3.5 × 10^5^ cells/well. After 24 h, they were pretreated with four triterpenes (5 μM) in a serum-free culture medium for 24 h. Then, 500 μM TBHP was added to each well. After 3 h, an MTT assay was conducted to evaluate the cytotoxicity of samples.

#### 2.3.2. Measurement of Intracellular Reactive Oxygen Species Production

ROS levels were measured using a DCFH-DA fluorescent probe as previously described [13]. Fluorescence intensity was recorded using a fluorescence spectrophotometer (Perkin-Elmer, Norwalk, CT, USA) every 10 min for 2 h with an excitation wavelength of 485 nm and an emission wavelength of 530 nm.

#### 2.3.3. Measurement of Hepatic Alanine Aminotransferase and Aspartate Aminotransferase Activities

Alanine aminotransferase (ALT) and aspartate aminotransferase (AST) activities were measured using ALT and AST assay kits (Biovision, CA, USA), according to the manufacturer’s instructions. Absorbance was measured at 570 and 450 nm for ALT and AST activities, respectively, using a microplate reader.

### 2.4. EA.hy926 Cell Culture

#### 2.4.1. Cell Culture and Cytotoxicity

EA.hy926 cells were seeded in 96-well plates at a density of 7.0 × 10^4^ cells per well. After 24 h, cells were pretreated with four triterpenes (5 μM) in a serum-free culture medium for 2 h. The culture medium containing the compound was discarded, and cells were treated with 500 μM H_2_O_2_ with or without compounds for 24 h. Then, an MTT assay was conducted to evaluate compound cytotoxicity.

#### 2.4.2. Measurement of Intracellular Reactive Oxygen Species Production

Intracellular ROS formation was quantified using a DCFH-DA fluorescent probe, as previously described [13]. The experimental procedure was the same as described above.

#### 2.4.3. Measurement of Reduced Glutathione and Lipid Peroxidation

EA.hy926 cells were seeded in 6-well plates at a density of 2.0 × 10^5^ cells per well. Total intracellular GSH was determined according to the method described by Baker, with certain modifications [14]. Lipid peroxidation was quantitated by measuring MDA levels using the thiobarbituric acid-reactive-substance assay [15]. 

#### 2.4.4. Nitric Oxide Measurement

EA.hy926 cells were seeded in 96-well plates at a density of 5.0 × 10^4^ cells/well. Cells were pretreated with compounds (10 μM) for 12 h and stimulated as described above, and supernatants were collected to measure NO production. Nitrite accumulation in supernatants was determined using the Griess reaction. An amount of 100 μL Griess reagent was added to 100 μL of supernatant. After incubating for 10 min at 37 °C, the absorbance of each mixture was measured at 550 nm using a microplate reader, and nitrite concentration was determined by comparison to dilutions of a sodium nitrite standard solution.

### 2.5. RAW264.7 Cell Culture

#### 2.5.1. Cell Culture and Cytotoxicity

RAW264.7 cells were seeded in 96-well plates at a density of 1 × 10^5^ cells/well. After 6 h, the culture medium was replaced with FBS-free DMEM containing LPS (1 μg/mL) and compounds and incubated for 18 h.

#### 2.5.2. Nitric Oxide Assay

RAW264.7 cells were seeded in 96-well plates at a density of 1 × 10^5^ cells/well. Cells were treated with triterpenes (10 μM) and stimulated as described above, and the supernatants were collected to measure NO production using Griess reagent. An amount of 100 μL Griess reagent was added to 100 μL of supernatant. After incubating for 10 min at 37 °C, the absorbance of each mixture was measured at 550 nm using a microplate reader.

#### 2.5.3. Measurement of Tumor Necrosis Factor-α and Interleukin-6

Concentrations of tumor necrosis factor (TNF)-α and interleukin-6 (IL-6) in culture supernatants were determined with a Mouse TNF (Mono/Mono) ELISA Set and a Mouse IL-6 ELISA Set (BD Bioscience, San Jose, CA, USA), in accordance with the manufacturer’s instructions. RAW264.7 cells were seeded in 96-well plates at a density of 1 × 10^5^ cells/well. Cells were treated with triterpenes (10 μM) and stimulated as described above, and supernatants were collected to measure pro-inflammatory cytokine production. Absorbance was measured at 450 nm with correction at 570 nm using a microplate reader.

### 2.6. Statistical Analysis

All data were expressed as means ± standard deviation (SD) and were representative of three independent experiments. Statistical analysis was conducted using one-way analysis of variance (ANOVA), followed by Duncan’s test using SAS version 9.4 (SAS Institute Inc., Cary, NC, USA). A value of *p* < 0.05 was taken as the criterion for statistical significance. Principal component analysis (PCA) was performed using PAST 4.03 software (Natural History Museum, University of Oslo, Norway). Hierarchical clustering analysis (HCA) was performed using R studio statistical software (version 2022.02, R Studio, Boston, MA, USA) for heatmap visualization.

## 3. Results and Discussion

### 3.1. Effects of Triterpenes on UVB-Exposed Hs68 Cells

**Figure 2:**
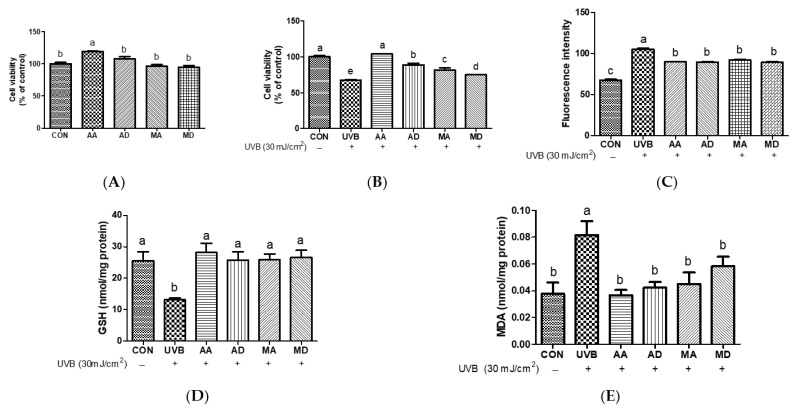
Effects of triterpenes (10 μM) on cell viability (**A**), protective effect (**B**), UVB-induced reactive oxygen species production (**C**), GSH depletion (**D**), and MDA production (**E**) in Hs68 cells. Values are expressed as means ± SD (*n* = 3). Different letters indicate significant (*p* < 0.05) differences based on Duncan’s multiple range test level. CON, control; UVB, ultraviolet B; GSH, glutathione; MDA, malondialdehyde; AA, asiatic acid; AD, asiaticoside; MA, madecassic acid; MD, madecassoside.

To examine the protective effects of triterpenes against UVB-induced skin damage, Hs68 cells were pretreated with AA, AD, MA, or MD (10 μM) for 24 h and irradiated with UVB. As shown in result, 10 μM triterpenes exerted no cytotoxic effects on Hs68 cells (Figure 2A and Figure S1). As shown in Figure 2B, UVB irradiation markedly decreased cell viability compared to control cells. Pretreatment with triterpenes significantly increased cell viability after UVB irradiation, with AA exhibiting the highest cytoprotective effect against UVB irradiation. To assess the impact of triterpenes on radical production in Hs68 cells, intracellular ROS levels were assessed in UVB-exposed Hs68 cells pretreated with vehicle, AA, AD, MA, or MD (10 μM) (Figure 2C). UVB irradiation significantly increases intracellular ROS production and pretreatment with triterpenes significantly ameliorated ROS production compared to that in vehicle-pretreated cells. No significant difference in ROS inhibition potency of tested triterpenes was observed. To assess intracellular non-enzymatic antioxidant defenses, alterations in intracellular GSH levels were examined in UVB-induced Hs68 cells after pretreatment with AA, AD, MA, or MD (10 μM) (Figure 2D). GSH levels decreased after UVB irradiation compared to those in control cells. All triterpenes similarly restored GSH levels to near the control level. We measured the intracellular concentration of MDA, an indicator of lipid peroxidation, in Hs68 cells. As shown in Figure 2E, UVB exposure significantly increased MDA concentrations, while pretreatment with triterpenes prevented this UVB-induced increase in MDA, showing significant differences compared with vehicle-treated cells. No significant differences were observed between treated samples. According to An et al., the treatment of human dermal fibroblasts with a titrated extract of *C. asiatica*, a reconstituted mixture comprising AA, MA, AD, and MD, showed a protective effect against UVB irradiation [16]. UVB radiation penetrates the skin and triggers ROS generation, activating pathways associated with skin aging. These ROS are responsible for the development of wrinkles and photoaging characteristics in UVB-induced skin aging [17]. GSH plays a crucial role in various physiological processes, including the scavenging of oxygen free radicals and the maintenance of intracellular redox balance. Previous studies have highlighted the role of GSH as an innate defense mechanism against UV-induced ROS production. GSH directly scavenges radicals through hydrogen transfer and acts as an essential factor for the enzyme GSH peroxidase, which further scavenges peroxide [18]. Repeated exposure to UV radiation generates peroxyl free radicals, which can attack unsaturated fatty acids in a lipid membrane, leading to the generation of MDA [19]. These results suggest that all triterpenes protect Hs68 cells against UVB-induced oxidative stress by suppressing ROS production and reducing levels of MDA and GSH.

**Figure 3:**
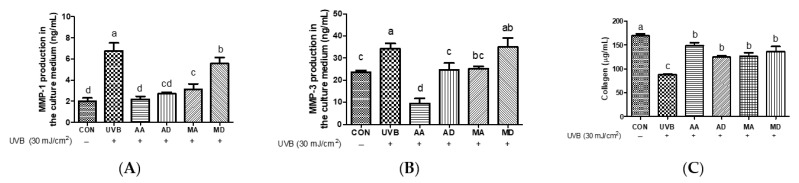
Effects of triterpenes (10 μM) on UVB-induced MMP-1 (**A**), MMP-3 (**B**), and collagen (**C**) production in Hs68 cells. Values are means ± SD (*n* = 3). Different letters indicate significant (*p* < 0.05) differences based on Duncan’s multiple range test level. CON, control; UVB, ultraviolet B; MMP-1, matrix metalloproteinase-1; MMP-3, matrix metalloproteinase-3; AA, asiatic acid; AD, asiaticoside; MA, madecassic acid; MD, madecassoside.

In a previous study, UVB irradiation was shown to generate lipid peroxidation products and hydroxyl radicals through the involvement of subsequent signaling cascades, ultimately triggering the upregulation of interstitial collagenase enzymes, such as MMP-1 and MMP-3, which are prominent members of the MMP family [20]. To investigate whether triterpenes suppress UVB-induced MMP-1 and MMP-3 secretion, we examined MMP production by UVB-treated Hs68 cells pretreated with AA, AD, MA, or MD. UVB irradiation promoted MMP-1 and MMP-3 production, whereas triterpenes depressed levels of MMP-1 and MMP-3, with a similar trend for both MMPs (Figure 3A,B). AA exhibited the most pronounced inhibition of UVB-induced MMP expression, whereas AD and MA showed similar levels of inhibition. However, MD did not show a discernible effect on MMP-3 inhibition and slightly reduced MMP-1 production compared to the UVB-only treatment group. To evaluate the effect of triterpenes on UVB irradiation-induced collagen reduction, collagen content was measured in UVB-induced Hs68 cells pretreated with AA, AD, MA, or MD. Total soluble collagen decreased markedly after UVB treatment, whereas pretreatment with triterpenes significantly ameliorated this decrease (Figure 3C). We hypothesize that the prominent inhibitory effect of AA on collagen degradation is mediated by MMP inhibition. UV irradiation-induced oxidative stress prompts the activation of activator protein-1, leading to increased MMP expression and collagen degradation [21]. Previous research has indicated that the n-butanol fraction of *C. asiatica* lysate and the isolated compound AD exhibit inhibition of MMP-1 [22]. In addition, Yingngam et al. reported that microwave-assisted extraction of crude extract-enriched triterpenes from *C. asiatica* leaves inhibited MMP-3 and MMP-9 production in UVB-irradiated dermal fibroblasts [23]. AD has been documented to stimulate type-I collagen synthesis via Smad signaling independent of transforming growth factor-β receptor 1 kinase in human dermal fibroblast cells [24]. The dermal extracellular matrix primarily consists of type I collagen, with minor proportions of type III collagen, elastin, proteoglycans, and fibronectin. Collagen fibrils thus play a vital role in maintaining skin strength and elasticity [25]. These results show that AA pretreatment effectively decreases MMP-1 and MMP-3 levels and protects against collagen degradation induced by oxidative stress better than other triterpene pretreatments.

### 3.2. Effects of Triterpenes against Oxidative Stress in HepG2 Cells

**Figure 4:**
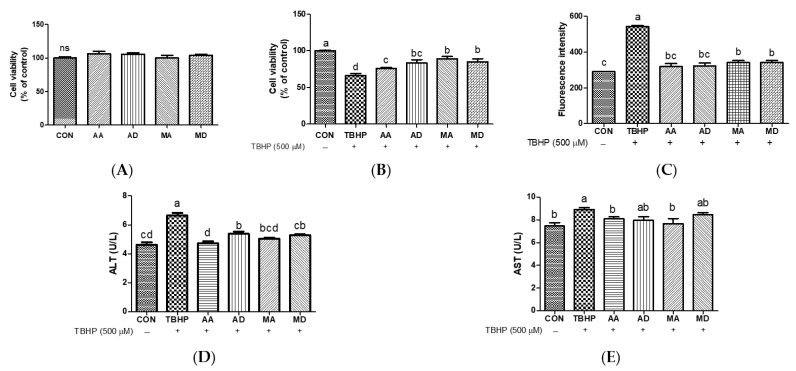
Effects of triterpenes (5 μM) on cytotoxicity (**A**), protective effects (**B**), reactive oxygen species production (**C**), ALT (**D**), and AST (**E**) in HepG2 cells. Values are expressed as means ± SD (*n* = 3). Different letters indicate significant (*p* < 0.05) differences based on Duncan’s multiple range test level. CON, control; TBHP, *tert*-butyl hydroperoxide; ALT, alanine transaminase; AST, aspartate aminotransferase; AA, asiatic acid; AD, asiaticoside; MA, madecassic acid; MD, madecassoside; ns, not significant.

To examine the protective effects of triterpenes against TBHP-induced hepatocyte damage, HepG2 cells were pretreated with AA, AD, MA, or MD (5 μM) for 24 h. None of these compounds exhibited cytotoxic effects on HepG2 cells (Figure 4A and Figure S2). As shown in Figure 4B, treatment with TBHP markedly decreased cell viability compared to vehicle, and all tested triterpenes significantly increased the viability of TBHP-treated cells. MA and MD increased cell viability more strongly than AA pretreatment. TBHP increased intracellular ROS generation, and all compounds markedly decreased ROS production (Figure 4C). To examine triterpene protective effects against TBHP-induced hepatocyte damage, ALT and AST levels were assessed in TBHP-treated HepG2 cells pretreated with AA, AD, MA, or MD. Treatment with TBHP significantly increased ALT and AST levels in comparison with vehicle control cells (Figure 4D,E). In contrast, pretreatment with all triterpenes significantly decreased ALT levels, and AA and MA pretreatments significantly decreased AST levels. Exposure of HepG2 cells to TBHP induces ROS generation, likely leading to apoptosis and cell death in HepG2 cells as a consequence of oxidative stress [26]. TBHP is a toxic compound that results in an increase in membrane permeability, leading to a disturbance in cellular membrane integrity [27]. Biochemical analysis of serum enzymes, including ALT and AST, has become the standard assay for hepatic injury [28]. Choi et al. reported that *C. asiatica* leaf extract significantly ameliorated increases in ALT and T-bilirubin compared to increases induced by dimethylnitrosamine, which can cause acute and chronic liver injury [29]. The *n*-butanol extract of *C. asiatica* protected hepatocyte cell viability against oxidative damage [30]. In a previous study, quercetin, the aglycone form of rutin, exhibited enhanced cytoprotective activity against oxidative stress compared to rutin, the glycoside form [31]. These findings indicate that TBHP-induced ROS generation is reduced by triterpene pretreatment, demonstrating that triterpenes may be potent ROS scavengers. Moreover, the aglycone forms of AA and MA effectively decreased ALT and AST levels during TBHP-induced oxidative stress in hepatocytes, whereas the glycoside forms AD and MD were relatively less effective compared to the aglycones.

### 3.3. Effects of Triterpenes on H_2_O_2_-Induced EA.hy926 Cells

**Figure 5:**
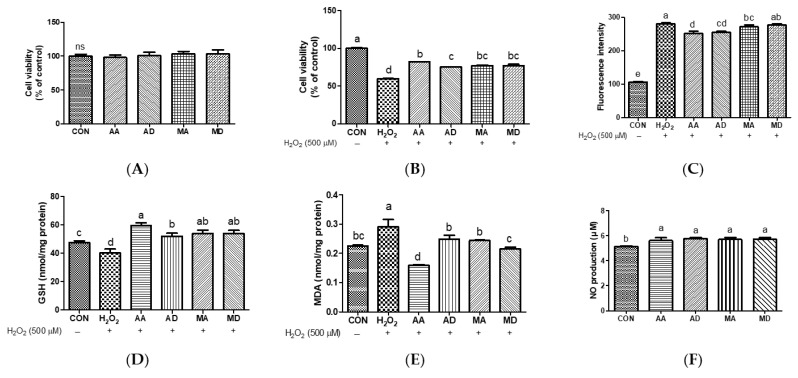
Effects of triterpenes (2.5 μM) on cell viability (**A**) and their protective effects (**B**) in H_2_O_2_-induced EA.hy926 cells. Effects of triterpenes (2.5 μM) on H_2_O_2_-induced ROS production (**C**), GSH depletion (**D**), and MDA production (**E**) in EA.hy926 cells. Effects of triterpenes (2.5 μM) on NO generation (**F**) in EA.hy926 cells. Values are expressed as means ± SD (*n* = 3). Different letters indicate significant (*p* < 0.05) differences based on Duncan’s multiple range test level. CON, control; H_2_O_2_, hydrogen peroxide; GSH, glutathione; MDA, malondialdehyde; NO, nitric oxide; AA, asiatic acid; AD, asiaticoside; MA, madecassic acid; MD, madecassoside; ns, not significant.

All triterpenes at a concentration of 2.5 μM exhibited no cytotoxic effects in EA.hy926 cells (Figure 5A and Figure S3). H_2_O_2_, one of the major types of ROS, has been reported to induce necrosis [32]. H_2_O_2_ markedly increased cell death compared to the control (Figure 5B), and all triterpenes significantly decreased H_2_O_2_-mediated cytotoxicity. AA showed the strongest protective effect. To evaluate whether triterpenes affect ROS production in EA.hy926 cells, ROS levels were measured (Figure 5C). Treatment with H_2_O_2_ markedly increases intracellular ROS production. Pretreatment with triterpenes AA, AD, and MA significantly reduced ROS production compared to cells treated with TBHP alone, whereas MD showed no effect. Among the triterpenes, AA most effectively inhibited ROS production. Decreased GSH levels were noted following H_2_O_2_ treatment compared to the control (Figure 5D). All triterpenes significantly prevented UVB-induced GSH depletion and restored GSH levels to near-basal levels. Treatment with H_2_O_2_ significantly increased MDA levels, and pretreatment with triterpenes decreased oxidative stress-induced MDA levels (Figure 5E). AA most potently inhibited GSH depletion and MDA production, whereas among the triterpenes, AD did not. As shown in Figure 5F, all tested triterpenes significantly increased NO concentration. H_2_O_2_ can permeate the plasma membrane and cause endothelial cell injury, and studies have indicated the involvement of ROS in endothelial cell apoptosis [33]. In a previous study by Bian et al., MD significantly increased H_2_O_2_-reduced cell viability in human umbilical vein endothelial cells [34]. NO is a critical effector molecule in the maintenance of vascular function that acts as an anti-atherosclerotic agent in the vasculature by diffusion into surrounding tissues. Declining NO production is a hallmark of endothelial cell dysfunction and predisposes individuals to the development of cardiovascular diseases. Methods to increase endothelial NO production are thus therapeutically desirable [35]. According to a previous study by Wang et al., AD significantly increases NO secretion in hypoxia-induced human pulmonary artery endothelial cells [36]. These results show that triterpenes, especially AA, alleviate ROS production, intracellular GSH depletion, and lipid peroxidation, supporting the maintenance of a normal cellular redox status and ameliorating oxidative damage. In addition, triterpenes can potentially protect vascular endothelial cells from oxidative stress by increasing NO production.

### 3.4. Effects of Triterpenes on LPS-Induced RAW264.7 Cells

**Figure 6:**
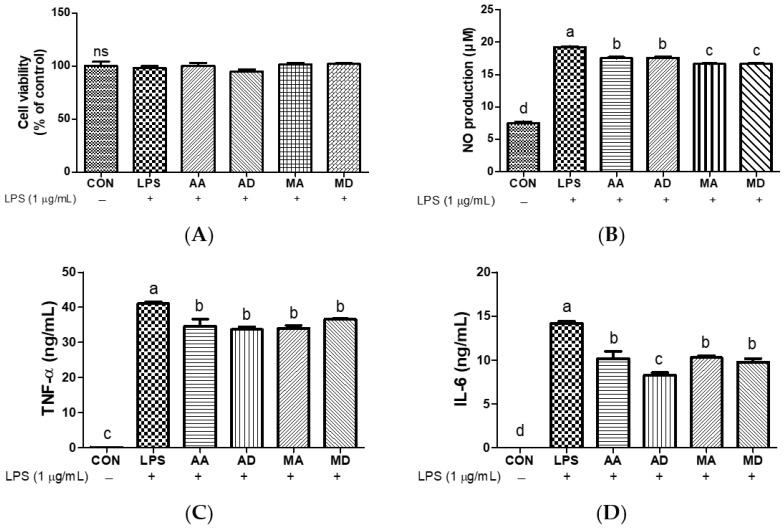
Effects of triterpenes (10 μM) on cell viability (**A**), NO production (**B**), TNF-α (**C**), and IL-6 (**D**) in LPS-induced RAW264.7 cells. Values are expressed as means ± SD (*n* = 3). Different letters indicate significant (*p* < 0.05) differences based on Duncan’s multiple range test level. CON, control; NO, nitric oxide; LPS, lipopolysaccharide; TNF-α, tumor necrosis factor-α; IL-6, interleukin-6; AA, asiatic acid; AD, asiaticoside; MA, madecassic acid; MD, madecassoside; ns, not significant.

To comparatively assess the antioxidant effects of triterpenes on LPS-induced oxidative stress, RAW264.7 cells were treated with AA, AD, MA, or MD (10 μM) in the presence of LPS (1 μg/mL). The tested triterpenes (10 μM) exhibited no cytotoxic effects in RAW264.7 cells (Figure 6A and Figure S4). LPS treatment significantly increased NO levels (Figure 6B), and all triterpenes decreased NO levels compared to the LPS group in RAW 264.7 cells, especially MA and MD. TNF-α and IL-6 levels in response to LPS-induced oxidative stress were quantified. TNF-α and IL-6 levels were markedly increased by oxidative stress, and treatment with triterpenes significantly decreased TNF-α and IL-6 production. A similar trend was observed for both cytokines, with AD exhibiting a particularly inhibitory effect on IL-6 (Figure 6C,D). NO plays a crucial role as an intracellular and intercellular signaling molecule that participates in the regulation of physiological processes in the immune system [37]. When NO interacts with superoxide, it generates peroxynitrite ions, exacerbating the inflammatory response [38]. Puttarak et al. reported that the anti-inflammatory efficacy of a *C. asiatica* extract enriched with pentacyclic triterpenes surpassed that of AD, MD, and MA individually, although it was lower than that of AA. This indicates that AA primarily inhibits NO production [39]. Other studies have indicated that AA and MA exhibit stronger inhibition of LPS-induced NO production than AD and MD [40]. Toll-like receptor ligands activate macrophages, directing the secretion of inflammatory cytokines, including TNF-α and IL-6, thereby amplifying both inflammatory and adaptive immune responses [41]. Our results show that AD, whose anti-inflammatory activity is well known, exhibits the highest inhibitory effect against LPS [42]. These results thus show that triterpenes effectively decrease NO, TNF-α, and IL-6 levels in LPS-induced oxidative stress.

### 3.5. Principal Component Analysis (PCA) and Hierarchical Clustering Analysis (HCA) of Triterpenes Based on Biological Activities

**Figure 7:**
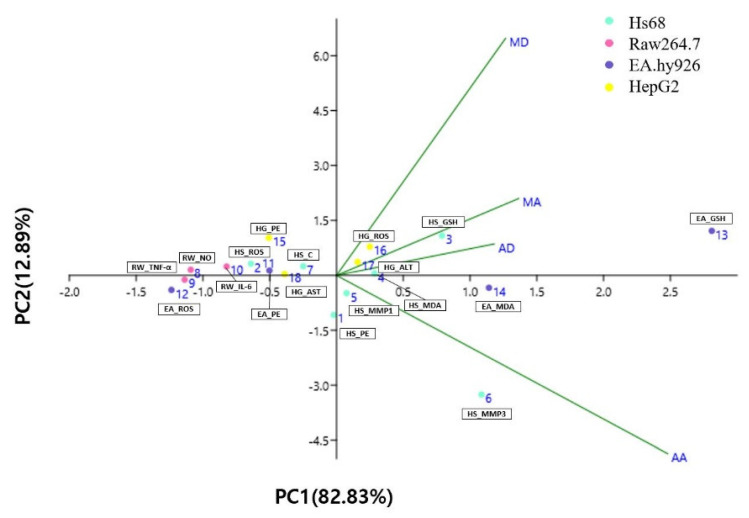
Two-dimensional scatter diagram of principal component analysis based on triterpene biological activities. AA, asiatic acid; AD, asiaticoside; MA, madecassic acid; MD, madecassoside; EA, EA.hy926 cells; HG, HepG2 cells; HS, Hs68 cells; RW, Raw264.7 macrophages; GSH, glutathione; ROS, reactive oxygen species; NO, nitric oxide; TNF-a, tumor necrosis factor-α; C, collagen; AST, aspartate aminotransferase PE, protective effect; IL-6, interleukin-6; MMP3, matrix metalloproteinase-3; ALT, alanine aminotransferase; MDA, malondialdehyde; MMP1, matrix metalloproteinase-1.

**Figure 8:**
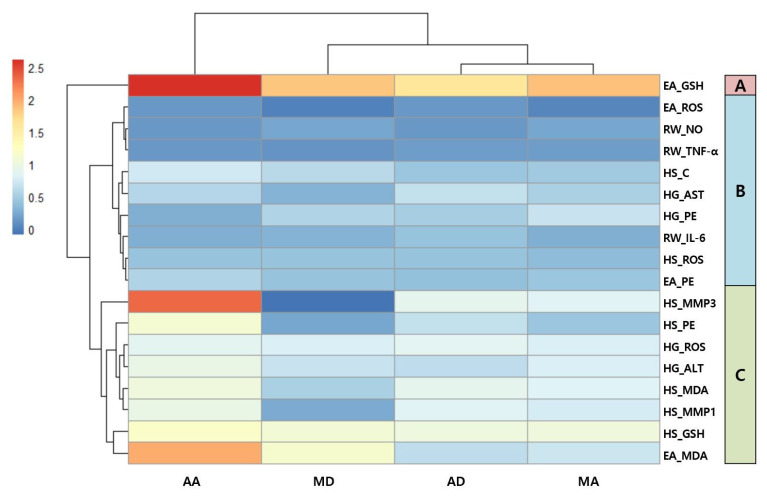
Hierarchical clustering analysis of triterpenes based on triterpene biological activities. AA, asiatic acid; AD, asiaticoside; MA, madecassic acid; MD, madecassoside; EA, EA.hy926 cells; HG, HepG2 cells; HS, Hs68 cells; RW, Raw264.7 macrophages; GSH, glutathione; ROS, reactive oxygen species; NO, nitric oxide; TNF-a, tumor necrosis factor-α; C, collagen; AST, aspartate aminotransferase PE, protective effect; IL-6, interleukin-6; MMP3, matrix metalloproteinase-3; ALT, alanine aminotransferase; MDA, malondialdehyde; MMP1, matrix metalloproteinase-1.

Principal component analysis (PCA) is a statistical technique classified under factor analysis that acts as a mathematical tool to capture the variability within a dataset [43]. PCA was used to characterize triterpene biological activity in Hs68, RAW264.7, HepG2, and EA.hy926 cells. Data were analyzed after calculating the percentage change compared with the stimulus group for standardization. Table 1 shows that PC1 differentiates the biological activities of AA, AD, MA, and MD. The first two PCs explain approximately 96% of the data variance and are plotted as biplots for PC1 and PC2 on the x- and y-axis, respectively (Figure 7). AA exhibits the largest loading, 0.75 PC1 (Table 1), and the contribution of PC1 to variability was the highest at 82.83%. Most of the data contributed significantly to PC1 and were distributed close to each other. Thus, the correlation between the two components is considered high. Hierarchical cluster analysis (HCA) examines the arrangement of samples into groups and subgroups and illustrates a hierarchical structure [44]. A heatmap is a graphical representation of a data matrix that visualizes cell values using a color gradient and offers a comprehensive view of both the highest and lowest values in the matrix [45]. The results of the HCA classification of the biological activities of triterpenes in Hs68, RAW264.7, HepG2, and EA.hy926 cells are displayed as a heat map. The data were analyzed after calculating the percent change compared with the stimulus group for standardization. The biological activities of triterpenes were classified into three groups based on their similarity (Figure 8). In group A, which included the GSH assay in EA.hy926 cells, all four triterpenes were effective, with AA being particularly effective. Group B includes biological activities that did not show distinct characteristics among the components, although they were effective. Group C includes assays in which AA clearly exerted the highest biological activity. Most of these assays were performed in Hs68 and HepG2 cells. As a result, the HCA results were in agreement with the results of the PCA, where AA is positioned at some distance from the others.

## 4. Conclusions

In conclusion, this study compared the biological activities of AA, AD, MA, and MD, the main triterpene components of *C. asiatica*, in skin fibroblasts, macrophages, hepatocytes, and endothelial cells. Statistical analyses showed that AA exerted the greatest overall influence on the examined variables and showed remarkable activity in Hs68 and HepG2 cells. In addition, these study results encourage further investigations in which aglycone forms are proportionally increased, because these forms show higher biological activities.

## Figures and Tables

**Table 1:** Principal component analysis of biological activities of triterpenes, with eigenvalues and percentage of variability explained by the first four components.

	PC 1	PC 2	PC 3	PC 4
AA	0.75	−0.58	−0.33	0.00
AD	0.36	0.10	0.64	0.67
MA	0.41	0.25	0.49	−0.72
MD	0.38	0.77	−0.49	0.15
Eigenvalue	0.89	0.14	0.04	0.00
Variability (%)	82.83	12.89	3.88	0.04
Cumulative variability (%)	82.83	95.72	99.60	100.00

AA, asiatic acid; AD, asiaticoside; MA, madecassic acid; MD, madecassoside.

## Data Availability

The research data that support this study will be shared upon reasonable request to the corresponding author. The data are not publicly available due to the funding conditions.

## Supplementary Materials

The following supporting information can be downloaded at https://www.mdpi.com/article/10.3390/antiox13040483/s1: Figure S1 Sample cytotoxicity in Hs68 cells; Figure S2. Sample cytotoxicity in HepG2 cells; Figure S3. Sample cytotoxicity in EA.hy926 cells; Figure S4. Sample cytotoxicity in RAW264.7 cells.
