# Supplementary material for: Comparison of the Antioxidant Potency of Four Triterpenes of Centella asiatica against Oxidative Stress
Source: Antioxidants (Basel). 2024 Apr 18;13(4):483. doi: 10.3390/antiox13040483 (PMC11047496; doi:10.3390/antiox13040483)
Supplement: Supplementary file 1 [file antioxidants-13-00483-s001.zip › antioxidants-2944968-supplementary.pdf]

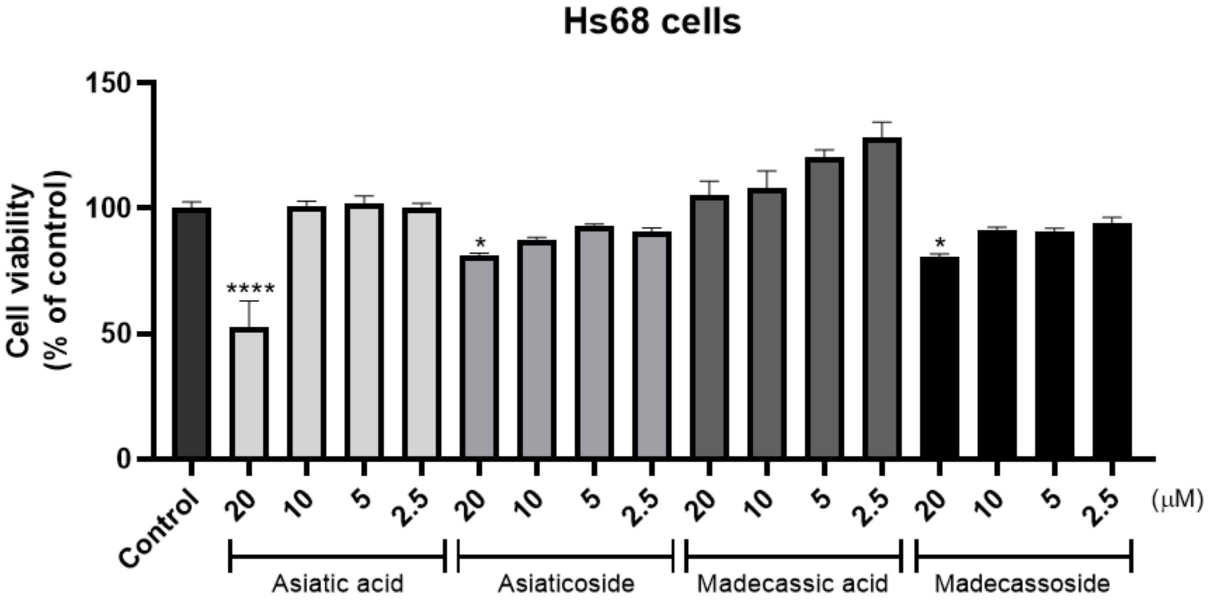

Figure S1. Sample cytotoxicity in Hs68 cells.

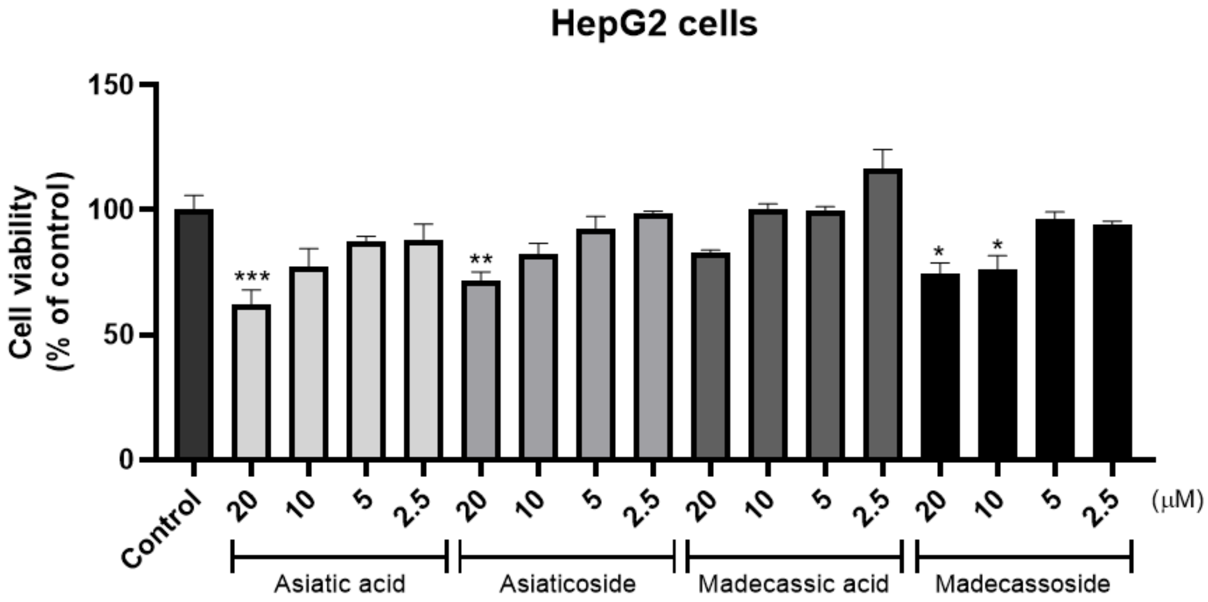

Figure S2. Sample cytotoxicity in HepG2 cells.

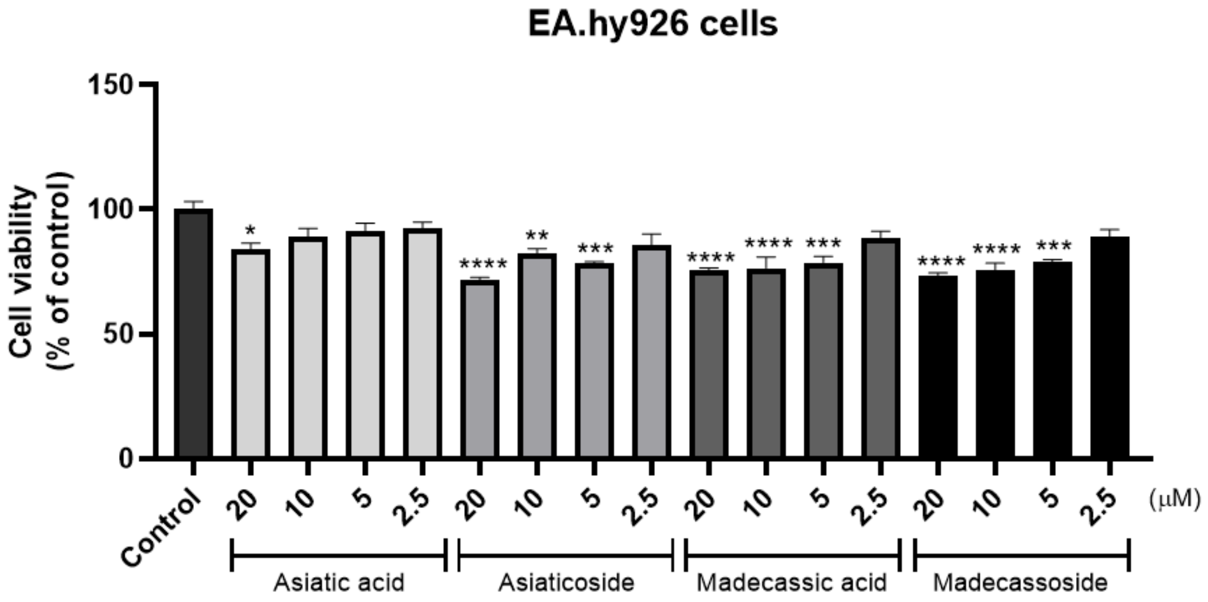

Figure S3. Sample cytotoxicity in EA.hy926 cells.

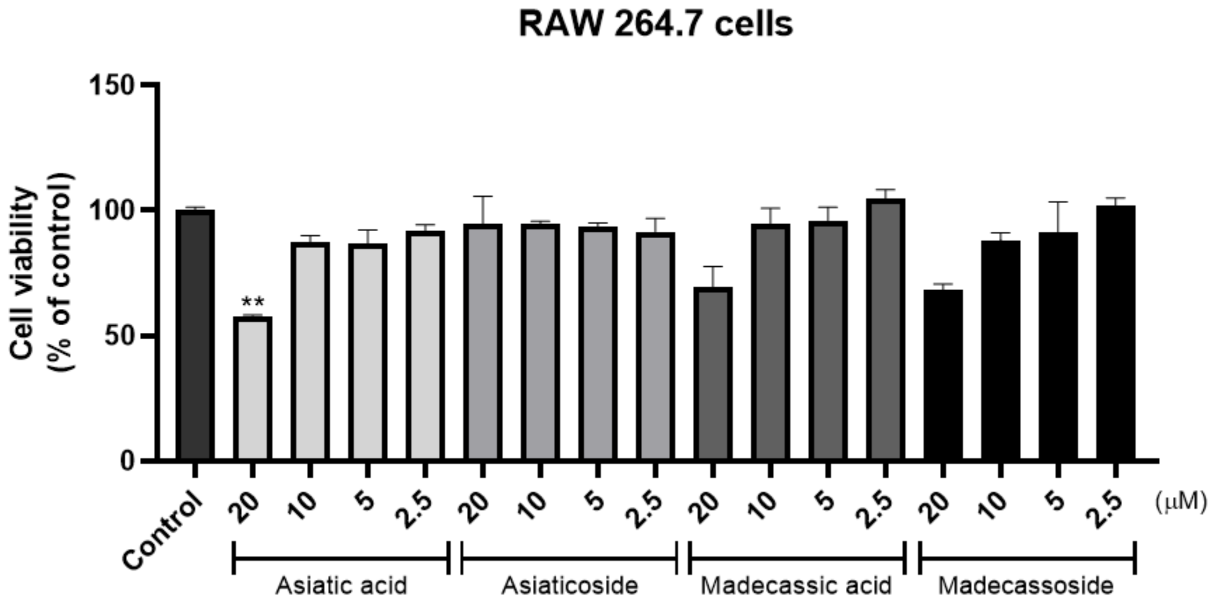

Figure S4. Sample cytotoxicity in RAW264.7 cells.
